# Unusual Morphology of a Distal Femur Fracture

**DOI:** 10.7759/cureus.70220

**Published:** 2024-09-25

**Authors:** Abdullah A Aldhibaib, Faisal A Alqifari, Abdulaziz M Almuhanna

**Affiliations:** 1 Orthopedics, Prince Sultan Military Medical City, Riyadh, SAU; 2 Orthopedics, King Saud University, Riyadh, SAU

**Keywords:** distal femur fracture, interarticular fracture, open reduction and internal fixation (orif), open reduction internal fixation, unusual fracture morphology

## Abstract

Fractures of the distal femur are rare but serious injuries that often follow a distinct pattern, occurring more frequently in both younger and older populations. In younger individuals, these fractures usually result from high-energy trauma, while in the elderly, they are often caused by domestic accidents.

A 65-year-old female with a history of type 2 diabetes, hypertension, anxiety, and dyslipidemia fell on the stairs, impacting her right knee. She experienced intense pain and was unable to walk. Examination showed a swollen right knee without abrasions, tenderness, or neurovascular compromise. X-rays revealed a displaced fracture of the medial femoral condyle, with a fracture line extending to the diaphysis, and a non-displaced lateral femoral condyle fracture. The case underscores a significant distal femur fracture in an elderly patient following a low-energy impact.

Early diagnosis and surgical stabilization are vital for good outcomes in femoral condylar fractures. Goals include anatomical reduction and restoring limb alignment while preserving vascularity. The choice of surgical methods depends on fracture configuration and surgeon preference. Surgery is the gold standard for displaced fractures.

## Introduction

Distal femur fractures are characterized by a bimodal distribution, primarily affecting two demographic groups: young men in their 30s, often due to high-energy impacts such as motor vehicle accidents, and older adults, especially women in their 70s or older, typically from low-energy falls at ground level [1]. Distal femur fractures account for less than 1% of all fractures and about 3%-6% of all femoral fractures, with 50% occurring in patients above 70 years [2,3]. According to a 2019 study conducted in Saudi Arabia, among 471 cases of femur fractures resulting from motor vehicle accidents, 64% were identified as mid-shaft fractures, 26% as proximal, and only 10% as distal femur fractures [4]. These fractures are relatively uncommon, making up just 0.4% of all fractures and 3% of those involving the femur; yet, they are serious, involving frequently comminuted or intra-articular fractures and often occurring in osteoporotic bone, which complicates surgical management [1].

In 1980, Seinsheimer introduced his classification system for distal femur fractures, dividing them into four types [5]. However, the AO/OTA (Association for Osteosynthesis/Orthopedic Trauma Association) system developed by Müller et al. is the most widely used classification for these fractures [6]. Treatment objectives follow AO foundation principles, focusing on achieving an anatomical reduction of the joint surface and restoring limb alignment, length, and rotation. Despite advancements in surgical approaches, the unique challenges of these fractures such as achieving stable fixation in osteoporotic and comminuted bone persist. Demographic shifts have shown a change in the gender ratio for distal femur fractures, with women now being more frequently affected than men, with a ratio of one man for every two women. Additionally, the average age at the time of fracture has increased, with a mean age of 61 years and more than half the cases occurring in individuals over the age of 65. This shift reflects broader aging population trends and underscores the need for tailored management strategies in the elderly population.

## Case presentation

**Figure 1:**
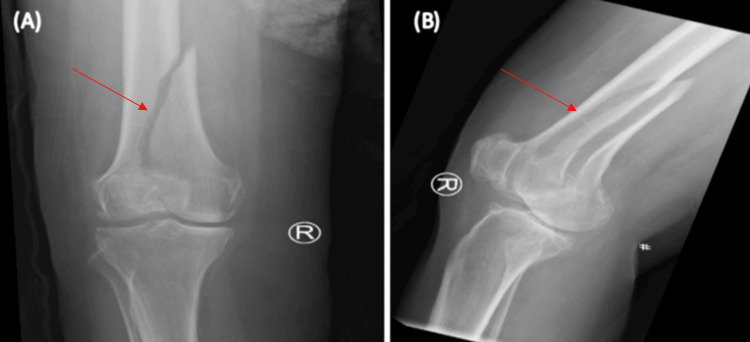
Initial X-ray upon presentation (A) An anterior-posterior (AP) view and (B) a lateral view. The initial X-ray upon presentation showed a displaced medial femoral condyle, with a fracture line from intercondylar notch extending to the diaphysis, and a non-displaced lateral femoral condyle.

A 65-year-old female with a medical history significant for type 2 diabetes mellitus, hypertension, generalized anxiety disorder, and dyslipidemia (all conditions being managed for over a decade) presented to the emergency department after a fall on the stairs. The incident involved a direct impact to her right knee. Post-fall, the patient reported an inability to walk accompanied by severe pain localized to the right knee. Upon examination, the patient was in a generally good condition. The examination of the right knee revealed noticeable swelling and tenderness over the distal femur, although there were no abrasions or ecchymosis. Importantly, the distal neurovascular status was intact. Radiographic evaluation, via X-ray, identified a displaced fracture of the medial femoral condyle. The fracture line extended from the intercondylar notch to the diaphysis. Additionally, a non-displaced fracture of the lateral femoral condyle was noted (Figure 1).

**Figure 2:**
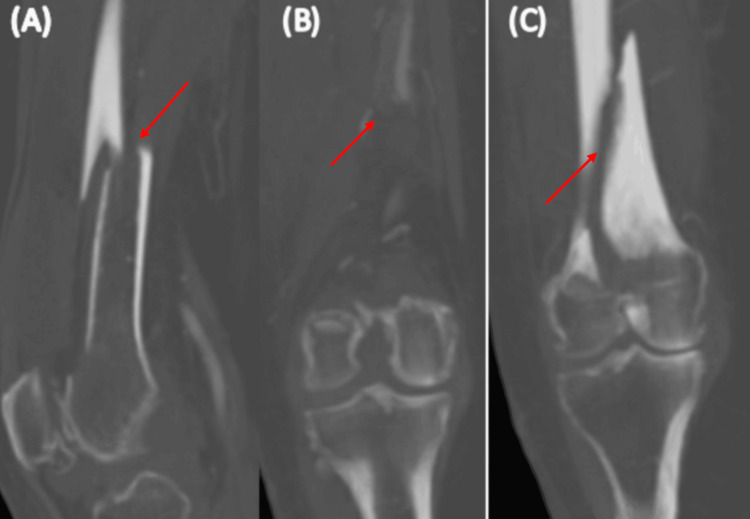
A computed tomography (CT) scan of the right femur Panel (A) is a sagittal view, while panels (B) and (C) are coronal views of the distal femur. There is a displaced longitudinal fracture line in the distal femur diaphysis extending into the joint surface, as shown by the arrow in panels (B) and (C). The fracture passes through the intercondylar notch, with the largest medial fragment displaced posteriorly, as shown by the arrow in Panel (A). The lateral femoral epimetaphysis shows an impacted fracture. This suggests a complex fracture involving both displacement and impaction, affecting the joint surface.

A computed tomography (CT) scan was done, which showed an AO classification of C1, but the medial femoral condyle was reaching the diaphysis (Figure 2).

## Discussion

Distal femur fractures, although rare, present significant clinical challenges, particularly in elderly populations. The case of a 65-year-old female patient, who sustained a distal femur fracture following a low-energy fall, exemplifies the complexities associated with managing such injuries in older adults, especially those with pre-existing health conditions. As highlighted in the introduction, distal femur fractures typically exhibit a bimodal distribution, affecting both younger, active individuals and the elderly. In this case, the patient presented with a fracture after a fall that is consistent with the characteristics of low-energy trauma more commonly seen in older adults. The evolving demographic trends, as noted in the literature, indicate that women are increasingly affected by these types of fractures, which is partly attributed to factors such as osteoporosis, a common concern in postmenopausal women [1].

**Figure 3:**
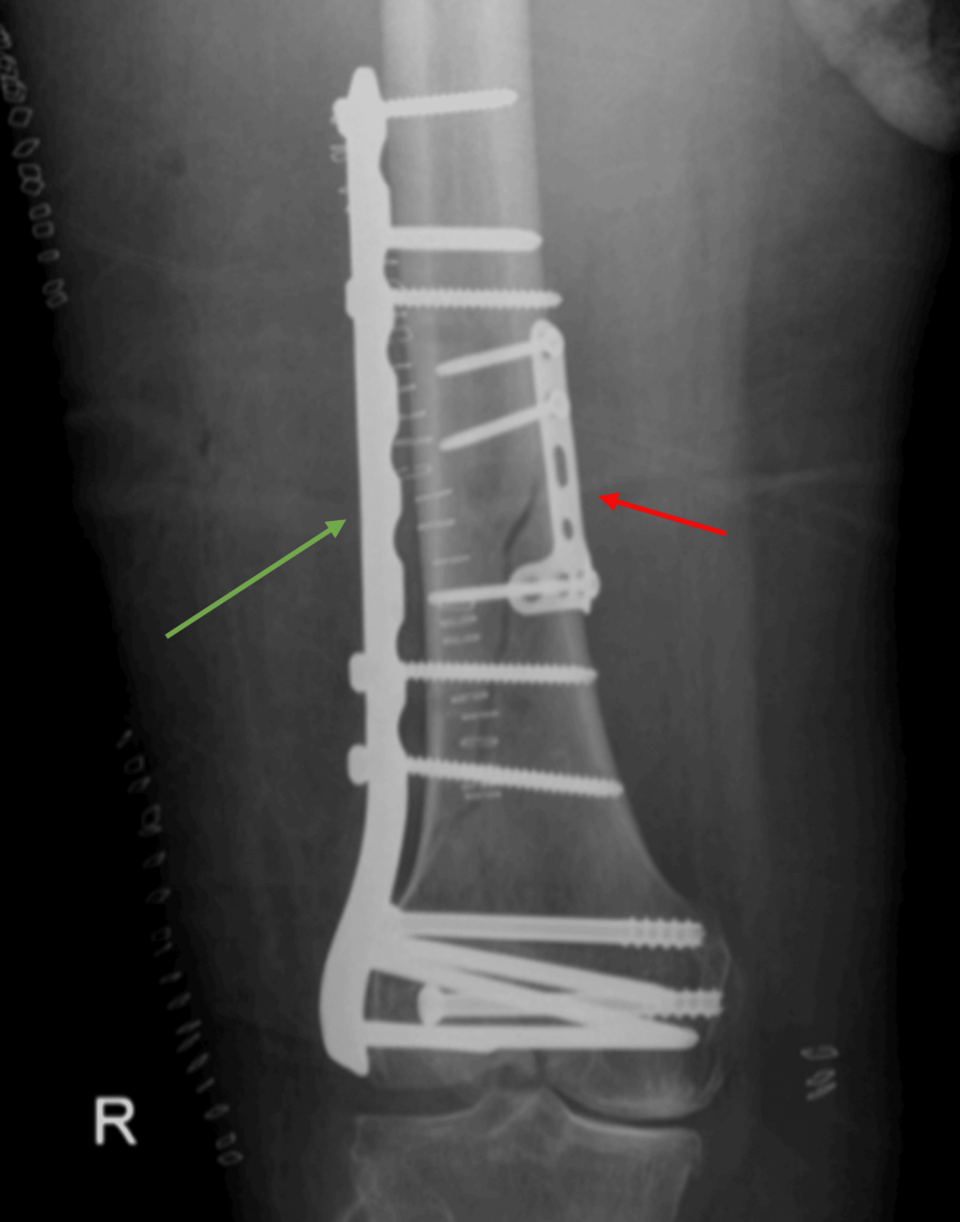
The immediate postoperative X-ray The postoperative X-ray displays an anterior-posterior (AP) view of the right femur. The T-plate has two screws inserted proximally and one screw placed distally, as indicated by the red arrow. The green arrow shows a lateral locking plate. The fixation resulted in a significant reduction of the fracture fragments.

The radiographic findings indicate a displaced fracture of the medial femoral condyle extending to the diaphysis, which is classified as AO type C1 based on the CT evaluation. This classification is significant, as it underscores the complexity of the fracture and the challenges presented in achieving stable reduction and fixation. The involvement of the medial femoral condyle and the extension of the fracture line complicate surgical decisions and highlight the need for precise surgical intervention to restore joint function and limb alignment. The surgical procedure was performed with the patient in a supine position on a radiolucent table, with a knee bump and a tourniquet applied. After prepping and draping the surgical area, closed reduction was achieved using pointed reduction clamps. Lag screws were then placed through the distal articular segment. An anteromedial approach was utilized, employing a four-hole T-plate, which biomechanically provides superior fixation compared to lag screws. Two screws were inserted proximally and one distally, followed by the placement of a lateral locking plate via a lateral distal femur approach (Figure 3).

**Figure 4:**
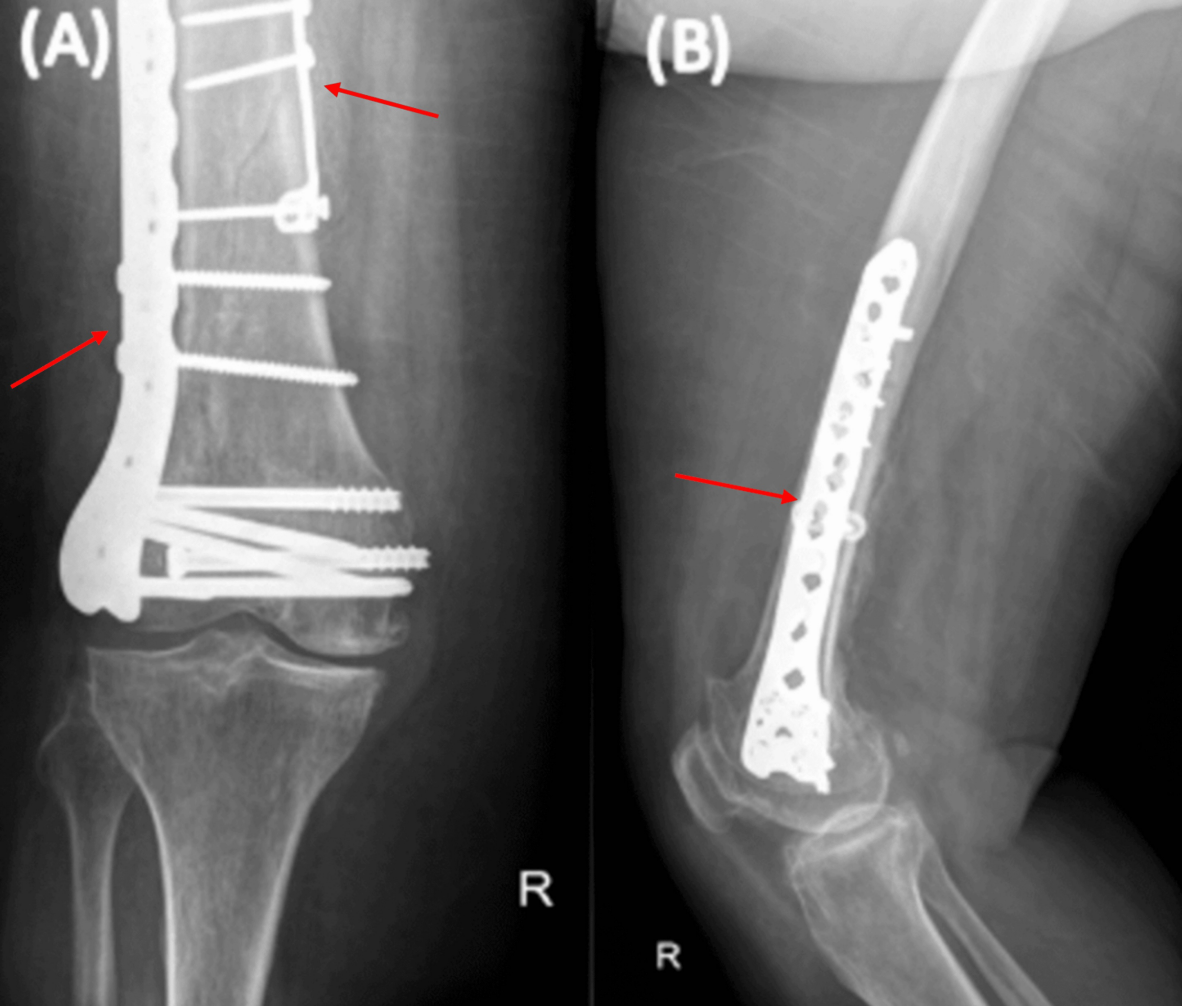
Three-month postoperative X-rays (A) AP and (B) lateral views of the right knee joint. Three-month postoperative photos show screws and plates (red arrows) used for internal fixation to stabilize the distal femur fracture. This technique is used to ensure proper alignment and healing of the bone after a complex fracture.

Closure was performed in layers. The patient was kept non-weight-bearing for six weeks, with immediate initiation of range of motion (ROM) exercises. The postoperative period was uncomplicated, with the wound fully healed after two weeks and skin clips removed. The patient was advised to continue non-weight-bearing activities and ROM exercises. A follow-up after six weeks revealed good signs of union, and weight-bearing was initiated as tolerated. A follow-up at three months indicated ongoing healing, with the patient mobilizing pain-free and expressing satisfaction with the outcome (Figure 4).

**Figure 5:**
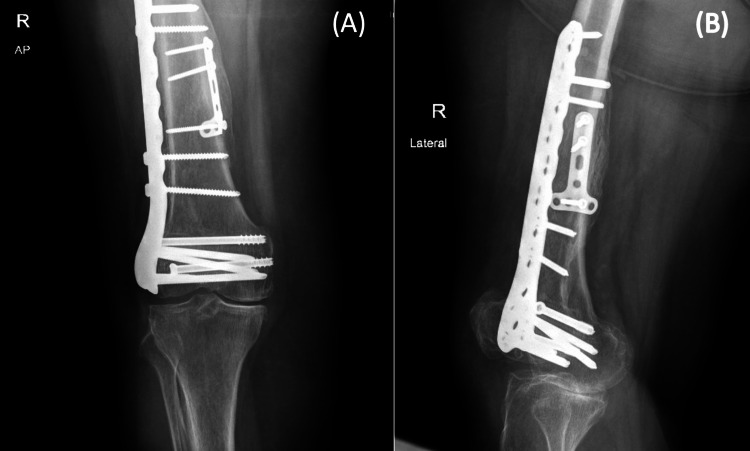
One-year follow-up X-rays (A) AP and (B) lateral views of the right knee joint. The one-year follow-up images show the healed fracture and proper alignment of the plate and screws.

A follow-up after one year also indicated good healing, the patient reported pain-free mobility and expressed satisfaction with the outcome (Figure 5). Surgical management of distal femur fractures aims for anatomical reduction and rigid internal fixation for early mobilization [7]. However, the presence of osteoporotic bone in elderly patients can substantially hinder the effectiveness of surgical fixation methods, as osteoporotic bones may not provide sufficient support for hardware used in fixation [8]. The choice of surgical exposure and implants typically depends on fracture configuration and surgeon preference, and accurate reduction is essential for successful outcomes [9]. This is particularly concerning in this patient, who has a history of chronic health issues that could further complicate the healing process. Thus, individualized treatment planning is critical, considering her overall health status and potential for recovery. The shift in the average age and changing gender ratios for these injuries must also be recognized in the management strategy. Understanding that the elderly population is at a higher risk for complications such as delayed healing and postoperative fractures is pivotal. The presence of comorbidities such as diabetes, hypertension, anxiety, and dyslipidemia also raises concerns regarding the patient’s ability to engage in rehabilitation and adhere to postoperative care protocols.

## Conclusions

The case of this 65-year-old female patient provides valuable insights into the management of a particular fracture pattern and the method used for the reduction and fixation of distal femur fractures within the aging population, underscoring the necessity for comprehensive evaluations and individualized treatment plans that consider patient demographics and comorbidities to achieve optimal outcomes. Early diagnosis and timely surgical stabilization are crucial for favorable results in femoral condylar fractures, with the key objectives including anatomic reduction of the articular surface and restoration of limb alignment, length, and rotation while preserving vascularity. The choice of surgical exposure and implants typically depends on fracture configuration and surgeon preference, and accurate reduction is essential for successful outcomes. Continued research and clinical focus are imperative for refining management strategies and enhancing surgical techniques for these complex injuries, as surgery remains the gold standard for managing severely displaced fractures with varying degrees of comminution.
